# The Common Combination of Aortic Stenosis with Mitral Regurgitation: Diagnostic Insight and Therapeutic Implications in the Modern Era of Advanced Echocardiography and Percutaneous Intervention

**DOI:** 10.3390/jcm10194364

**Published:** 2021-09-24

**Authors:** Francesca Mantovani, Andrea Barbieri, Alessandro Albini, Niccolò Bonini, Diego Fanti, Simone Fezzi, Martina Setti, Andrea Rossi, Flavio Ribichini, Giovanni Benfari

**Affiliations:** 1 Division of Cardiology, Azienda USL–IRCCS di Reggio Emilia, 42122 Reggio Emilia, Italy; francy_manto@hotmail.com; 2 Division of Cardiology, Department of Diagnostics, Clinical and Public Health Medicine, Policlinico University Hospital of Modena, University of Modena and Reggio Emilia, 41121 Modena, Italy; olmoberg@libero.it (A.B.); alessandro.albini@unimo.it (A.A.); niccolo.bonini@unimo.it (N.B.); 3 Section of Cardiology, University of Verona, 37129 Verona, Italy; diego.fanti@univr.it (D.F.); simone.fezzi@univr.it (S.F.); martina.setti@univr.it (M.S.); Andrea.rossi@univr.it (A.R.); flavio.ribichini@univr.it (F.R.)

**Keywords:** aortic stenosis, mitral regurgitation, combined heart valve disease, echocardiography, diagnosis, treatment

## Abstract

The combination of aortic stenosis (AS) and mitral regurgitation (MR) is common in patients with degenerative valvular disease. It is characterized by having complex pathophysiology, leading to potential diagnostic pitfalls. Evidence is scarce in the literature to direct the diagnostic framework and treatment of patients with this particular combination of multiple valvular diseases. In this complex scenario, the appropriate use of advanced echocardiography and multimodality imaging methods plays a central role. Transcatheter mitral valve replacement or repair and transcatheter aortic valve replacement widen the surgical options for valve diseases. Therefore, there is an increasing need to reconsider the function, timing, and mode intervention for patients with a combination of AS with MR towards more personalized treatment.

## 1. Introduction

In Western countries, degenerative aortic valve stenosis (AS) is commonly associated with mitral regurgitation (MR), as these are the most prevalent valvular heart diseases in the general population [1]. Despite the high prevalence of AS and MR, the majority of published research and most commonly used echocardiographic criteria have concentrated on either AS or MR as single valve lesions. Therefore, there is a scarcity of evidence in the literature to direct patients’ diagnostic frameworks and treatments with this particular combination of multiple valve heart disease. On the other hand, combined AS and MR pose a unique hemodynamic interaction and diagnostic challenge to the clinician in evaluating the effect of the lesions on ventricular function, cardiac remodeling and the timing of surgical or percutaneous intervention [2]. This article aims to provide a current review of severe degenerative AS combined with MR (AS + MR) concerning the primary role of echocardiography, with an emphasis on the role of advanced imaging and the complexity of the decision-making process. It is beyond the scope of this review to discuss AS + MR in the context of congenital heart valve diseases or other acquired pathogeneses including rheumatic heart disease and endocarditis.

## 2. Cause and Epidemiology

The mitral annulus, leaflets, and subvalvular apparatus frequently calcify to varying degrees in patients with AS, but the left ventricular (LV) size and function are typically normal. As a result, the conventional definition of secondary MR is rarely encountered, particularly in the elderly population [3]. In a prospective study focused on the MR mechanism in the context of AS, the left ventricular longitudinal function (reflecting the burden of left ventricular remodeling) was found to be the most important MR determinant; the tenting area and coaptation depth showed almost no role [4]. Likewise, for patients with AS and LV dysfunction, whether caused by ischemic heart disease or the consequences of chronic pressure overload, AS and LV dysfunction could contribute to the unbalancing between tethering and closing forces, almost always with a certain degree of mitral leaflets or annular calcification (mixed etiology) [5,6]. Diastolic dysfunction may also cause left atrial dilation, atrial fibrillation, and mitral annular dilation [7]. Primary MR may occur in patients with AS less frequently due to mitral valve prolapse. Although AS + MR due to chordal rupture are uncommon, they are generally associated with poor LV performance [8]. In a nationwide study in Sweden, 36,319 patients were discharged with an AS diagnosis based on the International Classification of Diseases-10 codes; 5.1% of these patients also had MR [9]. The prevalence of moderate or severe MR is even higher at the time of the aortic valve replacement (AVR). Significant (moderate or severe) MR is present in ∼15% of patients undergoing transcatheter aortic valve replacement (TAVR) [10,11,12]. More than half of them have organic MR (57% vs. 43% with functional MR) [13]. Among more than 600,000 patients undergoing valvular surgery included in the Society of Thoracic Surgeons database, 11% had multiple-valve procedures, of whom 58% underwent aortic and mitral valve surgery [14].

## 3. Pathophysiological Considerations and Diagnostic Insights

**Figure 1:**
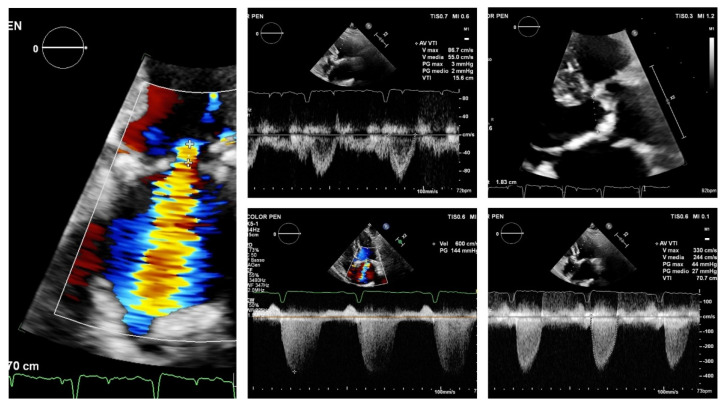
An elderly woman with low-gradient severe AS associated with MR and preserved ejection fraction. The MPG is 27 mm Hg and the Vmax is 330 cm/s. The left ventricular outflow tract’s pulsed wave Doppler velocity-time integral and diameter are 15.6 cm and 1.8 cm, respectively. The calculated SVI is 40 mL (24 mL/m^2^) and AVA is 0.58 cm^2^. The EROA by the 2D-PISA method is 0.20 cm^2^, Rvol = 37 mL. Of note, the MR jet velocity is 6 m/s because jet momentum flux (which is flow • v or EROA • v2) determines the color Doppler jet area. A 6 m/s jet shows 44% larger than a 5 m/s jet through the same EROA. AS: aortic stenosis; MR: mitral regurgitation; MPG: mean transaortic pressure gradient; Vmax: maximal transaortic velocity; SVI: stroke volume index; AVA: functional aortic valve area; v: velocity. 2D: bi-dimensional echocardiography; PISA: proximal isovelocity surface area; RVol: regurgitant volume; EROA: effective regurgitant orifice area.

The combination of volume overload caused by significant MR, whether primary or secondary, and the reduced preload reserve caused by LV hypertrophy resulting from AS, has a net effect on reducing forward flow across the aortic valve and, hence, the aortic velocity and gradient (paradoxical low-flow, low-gradient AS with preserved ejection fraction) [15], leading to a possible underestimation of the severity of AS by Doppler echocardiography [16]. The use of dobutamine stress echocardiography in the particular setting of low-flow, low-gradient AS due to significant MR may fail to induce a significant increase in LV outflow and may not enable the confirmation of AS severity. Furthermore, AS and MR have opposing effects on LV systolic function. As a result, if LV ejection fraction alone is used to reflect systolic function, the presence of MR will make the early detection of LV dysfunction in patients with AS more difficult. Conversely, a multidetector computed tomography assessment of the aortic valve calcium score can help distinguish between true-severe and pseudo-severe low-flow, low-gradient AS (true-severe, >2000 Agatston units in men and >1200 in women) in both classic and paradoxical (with preserved ejection fraction) low-flow, low-gradient AS [17]. At the same time, because jet momentum flux determines the color Doppler jet area, the color Doppler jet size has a tendency to overestimate MR severity in severe AS due to an increase in the systolic transmitral pressure gradient, which frequently causes MR jet velocity to exceed 6 m/s (Figure 1) [18]. In this specific hemodynamic condition, the regurgitant flow rate and the regurgitant volume will be increased for any given mitral effective regurgitant orifice area (EROA), Table 1 [19,20].

These considerations underscore the importance of performing a proper evaluation of both lesions using various quantitative parameters, always keeping in mind that quantifying the severity of any valvular lesion necessitates not only a single number but an integration of various parameters [21,22].

## 4. Role of Advanced Echocardiography

**Figure 2:**
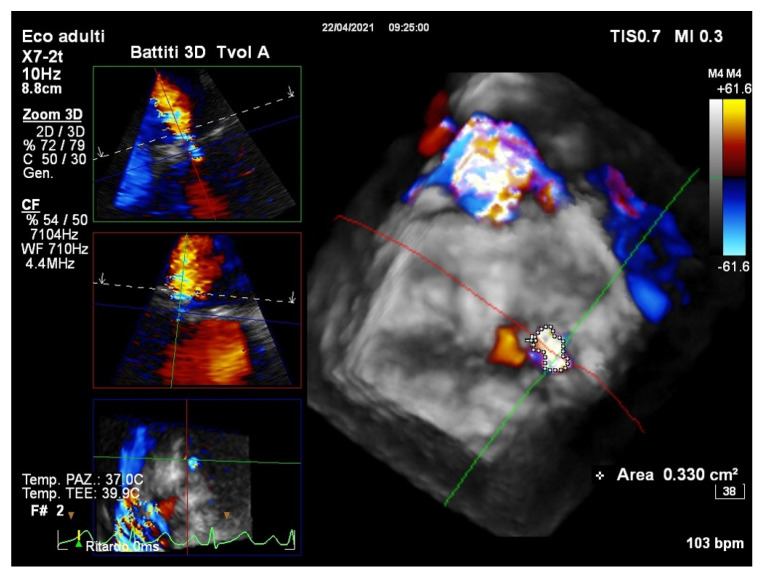
A RT3D color Doppler TEE dataset in a patient with severe AS and functional MR. The display of a 3D analysis software (Philips Medical Systems) showing a 3D view of the mitral valve and the MR jet from an LA perspective (right) and three reconstructed image planes in an orthogonal orientation of the MR jet: long-axis LVOT view (top left), 2-chamber view (middle left), short-axis view (bottom left) showing the asymmetric VCA (0.33 cm^2^ by direct planimetry). Since the formula assumes a relatively round orifice, the PISA by 2D echocardiography tends to underestimate secondary MR. EROA is often highly variable, even when holosystolic, so peak EROA (single frame showing the largest proximal flow convergence zone) can overestimate MR. Several recent studies compared 3D VCA measurements to other methods, especially for the quantification of MR, and found that the more asymmetric the VCA was, the better the accuracy of 3D measurements compared to 2D measurements. However, because of color Doppler blooming effects, the inclusion of low-velocity signals in the tracing, and the orifice’s nonplanarity, 3D VCA can overestimate EROA. Therefore, further clinical research is needed to determine new 3D VCA cutoff values. RT3D: real-time three-dimensional echocardiography; TEE: transesophageal echocardiography; MR: mitral regurgitation; AS: aortic stenosis; LA: left atrial; LVOT: left ventricular outflow tract; VCA: vena contracta area; PISA: proximal isovelocity surface area; 2D: bi-dimensional echocardiography; EROA: effective regurgitant orifice area.

The EROA and the vena contracta of MR are less afterload-dependent than the regurgitant volume and color-flow jet area, and better reflect MR’s true severity when associated with AS [9]. It is worth noting that currently, these parameters can be measured directly using real-time three-dimensional echocardiography (RT3DE) by the use of color Doppler, whether by transthoracic or transesophageal imaging [23,24,25], overcoming the limitations of conventional flow quantification using 2D color Doppler methods [26]. Recent research has validated the use of color Doppler RT3DE for EROA assessment based on the vena contracta area (VCA) and proximal isovelocity surface area (PISA) [27]. The routine use of color Doppler RT3DE to assess the VCA culminated in a paradigm change, with the majority of patients and etiologies now identifying the VCA as highly asymmetric (Figure 2) [28] Table 1.

**Figure 3:**
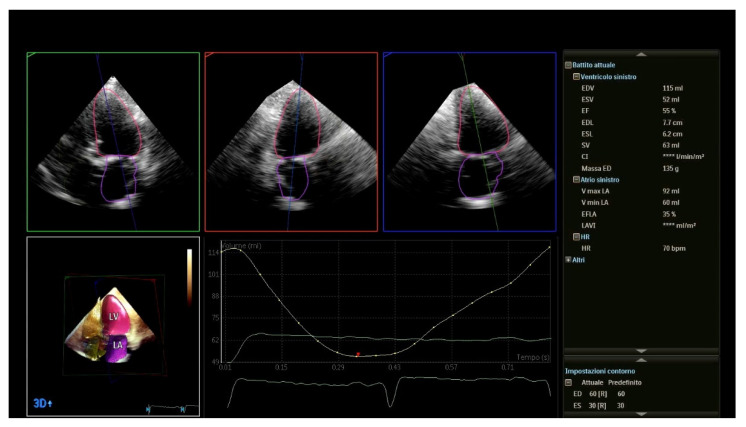
Three-dimensional measurements (LV volumes) obtained from automated DHM in the same patient. This advanced echocardiography allows for accurate, automated measurements of chamber volumes and function, providing larger and more accurate LV volumes with good agreement with cardiac magnetic resonance when analyzed using the default settings of the boundary detection sliders (end-diastolic default position = 60/60; end-systolic default position = 30/30). The calculated Rvol = SVLV – SVForward = 63 − 40 mL = 20 mL. EROA = Rvol/VTIRegJet = 20/186 = 0.10 cm^2^. LV: left ventricular; DHM: Dynamic Heart Model, Philips Healthcare, Andover, MA, USA; Rvol: regurgitant volume; SVLV: stroke volume ejected by the left ventricle; SVForward: forward stroke volume. 2D: bi-dimensional echocardiography; PISA: proximal isovelocity surface area; 3D: three-dimensional echocardiography; MR: mitral regurgitation; EROA: effective regurgitant orifice area.

Accurate measurement of VCA by RT3DE also improves the estimation of the MR flow volume, which is calculated by multiplying the VCA by the velocity-time integral of regurgitant flow by continuous-wave Doppler [29]. It could be argued that differences in EROA derived from 2D and 3D may be problematic. When color Doppler quantification methods are internally inconsistent, volumetric methods are recommended. Despite the lack of a “gold standard” for comparison, a growing amount of evidence points to an overestimation of functional MR with 2D-PISA compared to volumetric methods [30,31] (Figure 2). The drawback is that bi-dimensional echocardiography systematically underestimates LV volumes. Recent advances in artificial intelligence techniques, such as machine learning algorithms, have resulted in a highly feasible 3D analysis technique that detects LV boundaries automatically and allows for fast, accurate, and automated measurements of chamber volumes and function, providing larger and more accurate LV volumes with good agreement with cardiac magnetic resonance when analyzed using the default settings of the boundary detection sliders (Figure 3) [32].

## 5. The Consequences of Untreated Severe Mitral Regurgitation at the Time of Aortic Valve Replacement

Whereas isolated surgical AVR (SAVR) in old patients leads to acceptable mortality rate (approximatively 1–3%), the operative risk is substantially increased when double valve surgery is planned, with or without revascularization [33]. Conversely, although MR persistence after TAVR is seen in nearly half of patients with baseline significant MR, only 14.4% of this group remained highly symptomatic one month following TAVR [34]. Therefore, significant concomitant MR in severe AS is typically left untreated [13]. Studies looking at the outcomes of patients with MR that was left untreated at the time of SAVR did not show MR as a predictor of early or late mortality [5]. By contrast, a meta-analysis found that moderate-to-severe MR left untreated at the time of SAVR had worse early and late outcomes [35]. These inconsistencies are also evident for MR left untreated at the time of TAVR. Pooled results from two different meta-analyses have demonstrated a higher mortality rate at 30 days, 1 year, and 2 years following TAVR in patients with significant MR [36,37], whereas others did not [5]. These discrepancies may be due to distinct inclusion criteria; most studies used only qualitative grading of MR severity or failed to describe MR grading methodology. Only moderate MR was included in some studies [38], while moderate and severe MR were grouped in others [39,40,41]. The studies are further puzzled by a scarcity of detail regarding the mechanism of MR [10,42,43,44,45,46]; some included both primary and secondary MR [47,48,49], while others reported 100% secondary MR in AS [50,51], which is highly doubtful because, as previously stated, the mitral apparatus is usually calcified to some degree in patients with severe AS. As a result, these confounders severely restrict the conclusions that can be derived from the current literature. Besides, the clinical significance of functional MR in patients with AS has not been investigated quantitatively. Recently, our group showed that [52] the concomitant functional MR EROA >10 mm^2^ holds a higher risk during medical follow-up. Therefore, to better understand the effect of MR on mortality, future research with centralized core laboratories should standardize the assessment of MR severity and mechanisms [13]. Another possible explanation is that MR response to AVR, and not the baseline MR grade, affects the long-term prognosis following AVR. Recently, the SWEDEHEART registry showed that moderate/severe baseline MR in patients undergoing TAVR is associated with a mortality increase during the 5 years of follow-up. This risk is offset if MR improves to ≤mild, whereas the worsening of MR after TAVR is associated with a two-fold mortality increase [53]. Of note, symptoms resolution after TAVR despite MR persistence likely identifies a subset of patients in whom MR plays a more minor role in the overall morbidity burden than AS and is, thus, less likely to impact prognosis post-TAVR [34].

## 6. The Effects of Valve Replacement for Aortic Stenosis on Mitral Regurgitation

Usually, the resolution of AS causes a decrease in LV systolic pressure, which lowers the pressure gradient across the mitral valve and decreases the magnitude of MR. However, the mitral valve’s response to AVR is variable; although a favorable outcome has been documented in a large number of patients, in others, MR may remain unchanged or even worsen. For instance, in the Placement of Aortic Transcatheter Valves (PARTNER) trial, MR decreased in most patients after SAVR and TAVR (69.4% vs. 57.7%) but worsened in 2.8% and 5.8% of patients, respectively [39,54].

The mechanism by which LV pressure reduction following AVR does not reduce or even increase secondary MR is unknown. This is a significant issue because the ability to predict MR response to AVR can influence the treatment strategy in cases of AS + MR. According to echocardiographic studies, after TAVR, the 3D geometry of the mitral annulus does not change (i.e., it remains smaller than in controls) [55]. At the same time, mitral annulus calcification associated with leaflet restriction [56] and the degenerative etiology of MR are negative predictors of MR severity improvement, whereas the functional etiology of MR is a significant positive predictor of its improvement after TAVR. The resolution of the AS in secondary MR with mitral tethering will reduce the mitral tenting area in the acute phase, resulting in a decrease in MR severity [57]. Reversed LV chambers remodeling and the regression of LV concentric hypertrophy in the late postoperative period may potentially diminish MR weeks after surgery [58]. In addition, many other variables can predict improvement in secondary or mixed MR: dilated LV and a decreased ejection fraction (indicating a greater propensity for reverse remodeling), LV dyssynchrony due to new left bundle branch block, right ventricular pacing or ischemic wall motion abnormalities, and a significant decrease in the transaortic pressure gradient (including a high preoperative transvalvular pressure gradient) [59]. Moreover, secondary atrioventricular MR is common in patients with severe AS undergoing AVR [60]. Therefore, it is not surprising that the occurrence of atrial fibrillation, pulmonary hypertension, and atrial and/or mitral annulus dilatation, both of which are associated with higher chronic MR repercussions, has been linked to more limited improvement in MR [13]. Previous research found that patients who received a balloon-expandable valve had a higher rate of MR improvement (66.7%) than those who received a self-expandable valve (40.8%) [37]. The latter group has a higher rate of post-procedural left bundle branch block and pacemaker implantation, resulting in LV dyssynchrony and reduced mitral valve closing forces. Similarly, a significant paravalvular leak is associated with persistent MR [61]. Notably, prediction models based on these characteristics had poor discrimination abilities for predicting MR regression after AVR [34]. All together, these data suggest that it is currently difficult to estimate MR change in individual patients because there are no randomized trials, and no evidence-based guidelines about whether MR should be addressed during SAVR or TAVR. Balloon aortic valvuloplasty has been shown to reduce the severity of MR in nearly half of patients with severe AS and coexistent MR [62,63]. However, there is no evidence to support the regular use of this procedure to identify patients who do not need a second mitral procedure during AVR. Even the patient-prosthesis mismatch after AVR could limit the expected reduction of LV pressure and, thus, attenuate MR reduction. Moreover, it is also uncertain if SAVR or TAVR works better for reducing MR. Notably, with the advancement of bioprosthetic valve hemodynamic performance over the last few decades and the use of rigorous methods for defining patient-prosthesis mismatch, true-severe patient-prosthesis mismatch has become nearly obsolete in the current TAVR era [64].

## 7. Decision-Making and the Role of Heart Team

**Figure 4:**
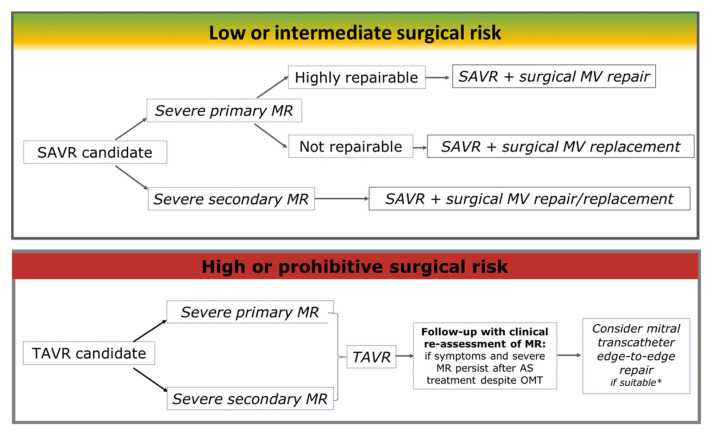
Management of patients with aortic valve stenosis and mitral regurgitation according to surgical risk.* Unsuitability for transcathether edge-to-edge mitral repair due to: (a) Severe mitral annular calcification with mitral stenosis or calcium extension into the leaflets, or restricted leaflet motion; (b) Severely calcified or fibrotic leaflet(s) (potential for mitral stenosis); (c) Prohibitively small mitral valve area (MVA < 3.5 cm^2^); (d) Severe mitral valve complexity (i.e., extensive Barlow’s disease with multiple jets; (e) excessive redundancy, calcifications, or scarcity of coaptation reserve in the leaflets; MR is primarily due to clefts); (f) Short or restricted posterior mitral leaflet (<5 mm in the intended grasping location). SAVR: surgical aortic valve replacement; MR: mitral regurgitation; MV: mitral valve; TAVR: transcatheter aortic valve replacement; AS: aortic stenosis; OMT: optimal medical therapy [21,73].

Current management guidelines for patients with multiple valve disease are based on limited data, as shown by the C-level of evidence for most recommendations [21,22]. Furthermore, TAVR is becoming the standard treatment option for elderly patients and those who are not at low surgical risk [21,22]. Furthermore, the availability of TAVR introduced the unique possibility, almost inconceivable with open heart surgery, to plan a second “staged” procedure on the mitral valve. However, there is a lack of knowledge on how to best handle patients with residual MR, taking into account the many different situations and complexities involved to arrive at the best solution for each case, which must be determined by a multidisciplinary team using joint decision-making with the patient. Nonetheless, a commonsense approach based on symptoms, AS severity classification, mechanism, and severity of MR has recently been proposed in the recently updated American College of Cardiology/American Heart Association guideline on valvular heart disease (Figure 4) [21]. As a general rule, patients with severe AS and severe MR are best treated with SAVR, and mitral valve repair in patients with low surgical risk [65]. If a patient has both AS and severe primary MR and the mitral valve cannot be repaired, the multidisciplinary team must make a decision about MR treatment, taking into account a variety of factors, including the additive risk of a mitral valve replacement [66]. Transcatheter mitral valve replacement or repair (TMVR/r) at a later date may be an option for these patients, but the outcome is likely to be suboptimal if the valve cannot be surgically repaired [67]. When a patient’s surgical risk is high or prohibitive, a staged procedure with TAVR, possibly followed by TMVR/r for patients whose MR did not regress, appears appealing [68]. However, the persistence of severe MR requires cautious clinical reassessment. If the patient presents asymptomatic, intervention may not be necessary considering that only a small proportion of patients awaiting TAVR are anatomically feasible for TMVR [69]. Indeed, one group has reported that the response to TMVR after TAVR may differ from that of patients without prior TAVR, due to TAVR-induced changes in mitral valve geometry [70]. However, it is equally true that the rapid increase in TMVR/r volume and the availability of more techniques or devices make this approach even more feasible [71,72]. Therefore, it will be essential to assess the short- and long-term outcomes of this strategy. Recently, the AMTRAC (Aortic+Mitral TRAnsCatheter) valve registry, a large international cohort of patients with significant residual MR after TAVR, found that TMVR/r following TAVR can be performed with a high procedural success rate and is associated with a substantial improvement in MR grade and New York Heart Association (NYHA) functional class after up to 1 year of follow-up [34]. However, randomized controlled trials are required to determine the real benefit of an additional mitral valve procedure compared to optimal medical therapy in patients with severe residual MR after SAVR or TAVR.

## 8. Conclusions

The combination of AS with MR is common among patients with degenerative valvular disease and is characterized by having complex pathophysiology, leading to potential diagnostic pitfalls that can be solved with careful echocardiographic examination or the appropriate use of advanced echocardiography and multimodality imaging methods. With the emergence of TMVR/r as a viable and often preferred treatment option for patients with AS, there is an increasing need to reconsider the function, timing, and mode of mitral valve intervention for patients with AS + MR. Personalized treatment at the time of AVR in these patients necessitates identifying independent predictors of MR progress and the management of the competing risk of post-discharge death. Currently, the principal evidence gaps in this issue concern the lack of validated predictive tools to better understand which patients with MR are likely to improve, the optimal timing of the intervention, and the applicability and the benefit of TMVR/r in patients with a persistence of functional MR following TAVR. Such a task can only be completed by collaborative efforts across multiple international sites and a properly designed prospective trial. Meanwhile, a multidisciplinary team should implement a case-by-case clinical management approach, considering various variables such as the personal risk profile, NYHA functional class, and the increased long-term morbidity of multiple prostheses.

## Figures and Tables

**Table 1:** Conventional echocardiographic parameters’ challenges and possible solutions multimodality and advanced imaging. MR: mitral regurgitation; AS: aortic stenosis; LVEF: left ventricular ejection fraction; LV: left ventricle; Rflow: regurgitant flow; Rvol: regurgitant volume; EROA: effective regurgitant orifice area; VC: vena contracta; PISA: proximal isovelocity surface area; RT3DE: real-time three-dimensional echocardiography; 3D: three-dimensional: CT, computed tomography.

Parameters	Conventional Echocardiographic Parameters’ Challenges
**Spectral Doppler signal**	MR spectral Doppler signal should not be mistaken for AS spectral Doppler signal.
**LV EF**	AS and MR have opposing effects on LV systolic function. Hence, the presence of MR will make early detection of LV dysfunction in patients with AS more difficult.
**Color Doppler jet size**	Color Doppler jet size tends to overestimate MR severity in severe AS due to an increase in the systolic transmitral pressure gradient, which frequently causes high MR jet velocities (>6 m/s).
**Rflow and Rvol**	In this specific hemodynamic condition, the regurgitant flow rate and the regurgitant volume is increased for any given mitral EROA.
**EROA**	EROA is prone to inaccuracy because of geometric assumptions of a circular orifice area and spherical PISA shell that are often invalid in secondary MR.
**Dobutamine stress echocardiography**	The use of dobutamine stress echocardiography in the particular setting of low-flow, low-gradient AS due to significant MR may fail to induce a significant increase in LV outflow and may not enable the confirmation of AS severity.
	**Possible Solutions Multimodality and Advanced Imaging**
**VC and PISA with color Doppler RT3DE for EROA and Rvol assessment**	Color Doppler RT3DE may identify cases of highly asymmetric VCA and PISA and therefore improve the estimation of EROA and MR flow volume.
**3D volumes for LVEF**	Three-dimensional volumes for LVEF are fast, accurate, automated measurements of chamber volumes and function providing more reliable RVol assessment
**CT aortic valve calcium score**	CT aortic valve calcium scores can help distinguish between true-severe and pseudo-severe low-flow, low-gradient AS (true-severe, >2000 Agatston units in men and >1200 in women) in both classic and paradoxical (with preserved ejection fraction) low-flow, low-gradient AS.
